# 

**Published:** 1877-12-20

**Authors:** 


					﻿£
Ph
w
Eh
Ph
w
Hl
<1
o
HI
o
w
s
Ph
P
O
pH-
o
Eh
fl
<1
fl
Ph
P
O
i-s
<n
Hl
M
Eh
Ph
O
32
S
HI
<1
fl
o
w
fl
Eh
Eh
fl
fl
32
fl
32
<1
fl
fl
Ph
vol, vn.
DECEMBER 20, 1877.
No. 12.
THE
SOUTHERN MEDICAL RECORD.
A MONTHLY JOURNAL OF PRACTICAL MEDICINE.
*-* QUICQUTD PR2ECIPIES ESTO BRETTS.”
w
o
fl
a I
e> I
O
g
g
a
fl
§
>
A
I—<
o
fl
02
>
fl
fl
fl
W
>
a
Hl
Q
£
l-H
A
fl
g
QQ
32
O
fl
Hl
Q
A
A
fl
JAS. P. HARRISON & CO., Pbintebs.
ATLANTA, GEOBGI A.
TERMS: $2.00 per Annum in Advance—Club Rates, 5 Copies for $8.00.
				

